# Supplementary material for: Influence of Ultra-Processed Foods Consumption on Redox Status and Inflammatory Signaling in Young Celiac Patients
Source: Nutrients. 2021 Jan 6;13(1):156. doi: 10.3390/nu13010156 (PMC7825019; doi:10.3390/nu13010156)
Supplement: Supplementary file 1 [file nutrients-13-00156-s001.pdf]

# Supplementary Material

**Supplement to:** Nestares et al. "Influence of ultraprocessed food consumption on redox status and inflammatory signalling in young celiac patients."

## Table of contents

**Supplementary table S1.** Differences in dietary habits in celiac children by percentage of energy intake from ultraprocessed foods (below 50% vs. above 50%).

| | **Ultraprocessed Foods Consumption** | | $p$ |
|---|---|---|---|
| | **Below 50% (n = 24)** | **Above 50% (n = 13)** | |
| **Food frequency** | | | |
| Refined cereals (s/month) | 82.8 (9.8) | 72.8 (13.4) | 0.552 |
| Potatoes (s/month) | 10.2 (2.2) | 12.2 (3.0) | 0.618 |
| Fruits (s/month) | 2.0 (1.5) | 1.4 (1.3) | 0.129 |
| Vegetables (s/month) | 108.4 (103.2) | 51.9 (30.3) | 0.077 |
| Pulses (s/month) | 13.0 (2.0) | 8.2 (2.7) | 0.171 |
| Fish (s/month) | 21.6 (2.4) | 18.4 (3.5) | 0.449 |
| Red meat and subproducts (s/month) | 46.5 (4.7) | 50.1 (6.4) | 0.653 |
| Poultry (s/month) | 19.7 (3.3) | 30.0 (4.4) | 0.072 |
| Whole dairy products (s/month) | 67.7 (49.4) | 24.8 (16.8) | 0.006 |
| Olive oil (s/month) | 68.7 (53.2) | 50.2 (23.2) | 0.301 |
| Sweets (s/month) | 51.6 (33.7) | 60.9 (35.8) | 0.362 |
| Nuts (s/month) | 29.4 (8.7) | 36.6 (11.9) | 0.629 |

Model adjusted for following a gluten free diet for at least 18 months. Data shown as mean (standard deviation); s, servings.
